# Clinical Society New York Polyclinic Medical School and Hospital

**Published:** 1912-03

**Authors:** 


### Clinical Society New York Polyclinic Medical School and Hospital.

Meeting of January 8, 1912.

STRANGULATED HERNIA OPERATED UPON UNDER COCAINE.

Case presented by Dr. Robert Brennan.

Patient was a man of 41 years of age and is single. His hernia was noticed at birth, and for sixteen years he wore a truss. At 16 he discontinued its use, but after four years became engaged in laborious work, and had to put it on again. One week ago the hernia came down, and, in spite of his efforts, he could not replace it. Three days after it came down he began to vomit, and this continued for two days, until he entered the hospital. He had a marked mitral lesion, which suggested the desirability of cocaine anesthesia. On cutting down, the sac enclosing the hernia was liberated, letting out bloody fluid, and a knuckle of black gut. Under hot applications to the gut, the color became restored in about an hour, and the wound was sewed up. His pulse promptly dropped from 140 to 100 after the operation and has remained normal since.

Dr. Morrow said he had been interested in cases of strangulated hernia from the fact that discoloration of the gut is often due to traction upon the vessels, and that time might be saved by promptly replacing the gut in the abdominal cavity. He had demonstrated that normal gut, when constricted at the femoral ring, for a short period of time, would promply lose its color and become black, and would regain it when shoved back into the abdomen.

THYRO-GLOSSAL CYST.

Case presented by Dr. J. A. Bodine.

Dr. Bodine showed a specimen of thyro-glossal cyst which he had removed from a case in its entirety. The case was somewhat uncommon. He had noticed from former careful observations with Dr. Myles that these cysts had a way of returning after operation, or at least leaving a suppurating condition in the neck. To obtain success, the cyst must be dissected from the base of the tongue to the thyroid gland, including the ducts. The cyst wall was thin, and nothing should be left if success was to be obtained and a cure promised.

Dr. Morrow said that he had never operated upon a thyroglossal cyst, but had a case of bronchial cyst which came under the class of congenital malformations. This case was very difficult because the wall of the fistula, which opened into the pharynx, was adherent to the deep vessels, and it was very hard to separate them, but this was finally accomplished.

AMPUTATION OF FINGER TIP, WITH REPLACEMENT AND RECOVERY OF FINGER.

Case presented by Dr. J. A. Bodine.

Dr. Bodine showed a case of a little fellow who, while working at a cloth-cutting device in a clothing shop, caught his finger tip in the machinery, cutting off the finger about half an inch from the tip. The boy was badly frightened, and had run to the hospital, leaving the finger tip on the floor in the shop. A friend was sent after it, and returned two hours later, bringing the tip, which was rather dried up but softened after soaking in normal salt solution. The tip was put in place and bound up. The finger showed complete healing and a round line of scar tissue where the junction had taken place.

RESECTION OF THE POSTERIOR ROOT OF THE TRI-FACIAL NERVE.

Case presented by Dr. J. A. Bodine.

Dr. Bodine showed a case of tri-facial neuralgia of long standing in a woman 60 years of age. She had tried injections of alcohol from time to time, but these finally failed to relieve her, and she was willing to have anything done. With the patient in a sitting position, Dr. Bodine had opend the skull, in front of the ear, just in front of the zygoma, the opening being 5 cm. in diameter, the bone removed and the brain lifted up. The tri-facial ganglion was not removed, but he went in behind it, and put a hook around the root, and resected it. Twenty-four hours after the operation she was entirely free from pain, and is cured for the balance of her life. There was no particular difficulty about the operation, although it was the first he had done of the kind; and, so far as he knew, was the first time it had been performed in this city. It was not removing the ganglion but the pulling out of the sensory root from the pons varolii which rendered the area very dangerous. It is not difficult, however, for anyone trained in surgery. He believed that he had separated the sensory from the motor roots, as mastication had not been interfered with. Such cases as had been treated by this method had been successful. At the beginning of the operation he had torn the middle meningeal artery, but there was no trouble checking the hemorrhage. Dr. Bodine thought that the fear of hemorrhage made cowards of many surgeons. The patient did not suffer from shock, and the pulse did not go above 90. The operation requires skill and judgment, as it is distinctly a one-man operation from start to finish.

Dr. Keller had given this case of Dr. Bodine's four alcohol injections, with slight benefit. Alcohol injections usually give relief in these cases, and, in his experience of fifty cases, he had had excellent results. Where relief was not obtained, he thought that the lesion was further back than the injection reached. Even when a resection was contemplated, Dr. Keller thought it benefited the patient to precede the resection by alcohol injection.

Dr. Connor welcomed any method of resection which spared the motor roots, as a ganglion resection usually resulted in a loss of the eye.

### CASE OF SALPINGITIS INFECTION FOLLOWING CONCEPTION.

Case presented by Dr. Morgan.

Dr. Morgan showed a case of a woman 31 years of age, married, with one child 11 years of age, and a history of several miscarriages. Her husband stated that she was exposed to gonorrhea, but she gave no history of inconvenience. Four weeks before admission to hospital she called her family physician, who found her in pain, and bleeding. She said she had missed one period, and was afraid she was going to miscarry. Bimanual examination showed a mass posterior to the uterus, and a diagnosis was made of a probable ruptured ectopic pregnancy. When seen a little later there was a tremendous mass. On consultation she had been removed to the hospital, and a laparotomy performed, and a pregnant uterus found. There was nothing to indicate a submucous, or an intra-mural fibroid. Further examination brought to light a tubo-ovarian cyst on the right side, and a chronic pyo-salpingitis of the left tube, with the fimbriated extremity closed. The tube and ovary on the right side were removed, and the left tube also. Dr. Morgan was amazed that the woman was pregnant. She began to menstruate after a rest of two weeks in bed. The interesting feature was that the woman became pregnant after an infection which apparently closed both tubes.

